# Topography and Lateralization of Nodal Metastases in Muscle-Invasive Bladder Cancer Using Super-Extended Pelvic Lymph Node Dissection with the Sentinel Lymph Node Technique

**DOI:** 10.3390/jcm13175127

**Published:** 2024-08-29

**Authors:** Adam Gurwin, Jakub Karwacki, Mateusz Dorochowicz, Kamil Kowalczyk, Łukasz Nowak, Diana Jędrzejuk, Wojciech Krajewski, Agnieszka Hałoń, Marek Bolanowski, Tomasz Szydełko, Bartosz Małkiewicz

**Affiliations:** 1 University Center of Excellence in Urology, Department of Minimally Invasive and Robotic Urology, Wroclaw Medical University, 50-556 Wroclaw, Poland; 2 Department of Endocrinology, Diabetes and Isotope Therapy, Wroclaw Medical University, 50-367 Wroclaw, Poland; 3 Department of Clinical and Experimental Pathology, Wroclaw Medical University, 50-556 Wroclaw, Poland

**Keywords:** muscle-invasive bladder cancer, sentinel lymph node, lymph node invasion, topography, mapping, lateralization, lymphadenectomy, PLND

## Abstract

**Background:** This study assessed the topography and lateralization of lymph node (LN) metastases in muscle-invasive bladder cancer (MIBC) patients using super-extended pelvic lymph node dissection (sePLND) with sentinel lymph node dissection (SLND). **Methods:** We analyzed 54 MIBC patients who underwent cystectomy with sePLND and SLND. Tumor location was classified using cystoscopy. Nanocolloid-Tc-99m was injected peritumorally. Preoperative SPECT/CT lymphoscintigraphy and an intraoperative gamma probe were used for SLN detection. **Results:** A total of 1414 LNs, including 192 SLNs, were resected from 54 patients. Metastases were found in 72 LNs from 22 patients (41%). The obturator fossa was the primary site for LN metastases (37.5%). SLNs were most common in the external iliac region (34.4%). In 36% of the patients with positive LNs, metastases were identified only through sePLND. In 9% of the patients, metastases were found solely in the pararectal region, identified through SLND. Tumor lateralization correlated with ipsilateral positive LNs, but 20% of the patients had contralateral metastases. **Conclusions:** The pararectal region may be the exclusive site for positive LNs in MIBC. The obturator fossa is the most prevalent region for LN metastases. Unilateral PLND should be avoided due to the risk of contralateral metastases. Combining sePLND with SLND improves staging.

## 1. Introduction

Bladder cancer (BCa) is the 10th most diagnosed cancer globally, with an estimated 573,000 new cases and 212,000 cancer-specific deaths annually. Worldwide, BCa is more common in men than in women, with men experiencing incidence and mortality approximately four times higher than those of women [1]. In recent years, a decrease in the mortality rate of BCa has been reported despite the increasing global incidence [2]. Muscle-invasive bladder cancer (MIBC) accounts for 30% of BCa cases. Lymph node (LN) metastases in addition to local tumor progression play a pivotal role as a primary prognostic factor. LN status significantly affects cancer-specific survival, even in the absence of other adverse prognostic factors [3]. Despite the best possible treatment and the absence of LN metastases, MIBC has a five-year mortality rate of 50%. This suggests that cancer metastases remain undetected by existing staging methods in half of MIBC patients [4,5].

Due to the insufficient sensitivity of non-invasive diagnostic methods in detecting LN metastases, the gold standard treatment for MIBC patients is radical cystectomy (RC) with bilateral pelvic lymph node dissection (PLND) [6]. However, the optimal extent of PLND remains an unresolved issue because the location of LN metastases varies among patients [7,8,9]. While PLND is the best nodal staging tool, its impact on oncological outcomes and the risk of perioperative comorbidities makes the overall benefit unclear [10]. Current guidelines emphasize that bilateral PLND should be an integral part of RC in MIBC [11,12]. Both the European Association of Urology (EAU) and the American Urological Association (AUA) advocate for dissecting the external iliac, internal iliac, and obturator LNs. This recommendation is based on evidence from major randomized trials, which indicate that more extensive PLND does not significantly improve survival outcomes [13,14]. The first LN or a cluster of LNs located in the direct path of lymphatic drainage from the primary tumor site is known as the sentinel lymph node (SLN). SLN mapping may help to determine the appropriate individual extent of PLND, thereby avoiding overtreatment.

The first objective of this study was to assess the precise topography of LN metastases in patients with MIBC through the utilization of super-extended PLND (sePLND) with SLN dissection (SLND). The second objective was to evaluate the lateralization of LN metastases in comparison to the intravesical location of the tumor. Finally, the last objective was to compare various PLND templates to the topography of LN metastases. The diagnostic value of the SLN technique itself was demonstrated in our previous study [15].

## 2. Materials and Methods

### 2.1. Patients and Study Design

**Figure 1:**
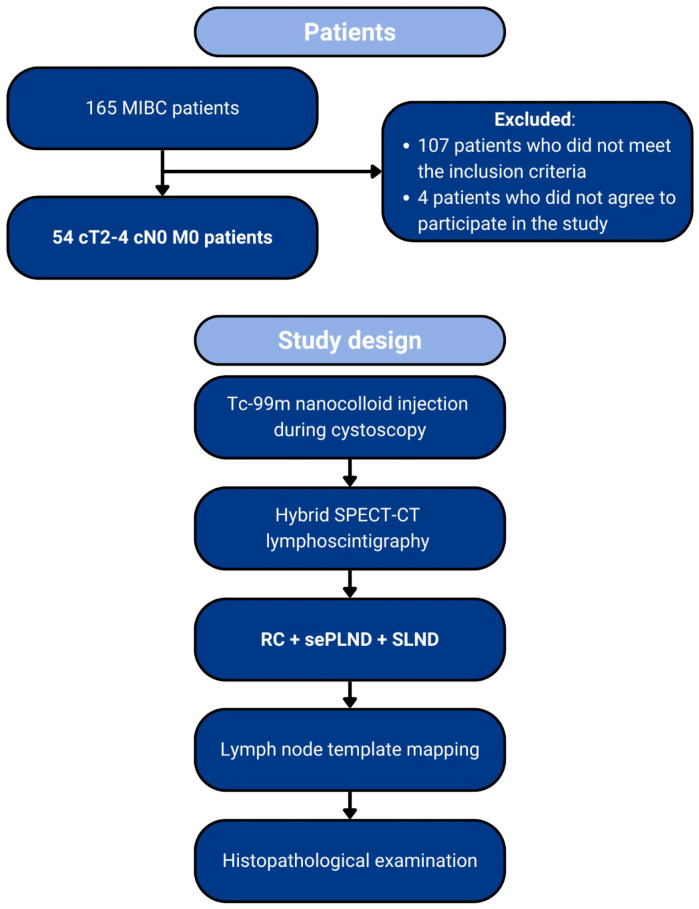
Patient selection and study design.

This research is part of a prospective intervention-based study conducted from March 2015 to October 2018 and involved 165 patients with MIBC who were scheduled to undergo radical cystectomy with PLND in a tertiary center. Of these patients, 54 met the study’s inclusion criteria and agreed to participate in the study. The inclusion criteria were as follows: (1) pathological stage pT ≥ 2; (2) no signs of LN metastases on contrast-enhanced CT or MRI scans (LNs with a short axis ≥ 8 mm); (3) no evidence of distant metastasis on contrast-enhanced CT or MRI; (4) possess a World Health Organization performance status of 0 or 1; and (5) absence of any known factors before surgery that could affect lymphatic drainage (e.g., pelvic surgery or pelvic radiotherapy). The exclusion criteria included: (1) inability or refusal to give informed consent; (2) any coagulation disorder; (3) inability to perform cystoscopy or SPECT-CT lymphoscintigraphy; and (4) existing contraindications for performing PLND. Neoadjuvant chemotherapy was allowed, and the surgeries were performed with curative intent. The intervention involved administering a radiocolloid, followed by hybrid SPECT-CT lymphoscintigraphy. The primary tumor’s staging was carried out using conventional cross-sectional imaging. The study was conducted in accordance with the Declaration of Helsinki and was approved by the Wroclaw Medical University Ethics Committee (protocol code: KB 29/2015; approval date: 29 January 2015). Before taking part in the study, all patients provided written consent after receiving detailed information about the research. An overview of the study design is presented in Figure 1.

### 2.2. Study Outcomes

The primary outcome of this study was to determine the precise topography of LN metastases in patients with MIBC using sePLND combined with SLND. The secondary outcomes included: (1) evaluating the lateralization of LN metastases in relation to the intravesical location of the tumor and (2) comparing the effectiveness of various PLND templates against the actual topography of LN metastases.

### 2.3. Radiocolloid Injection and SPECT-CT Imaging

On the day before the scheduled surgery, Tc-99m nanocolloid (Nanocoll; GE Healthcare, Milano, Italy) injections were administered during cystoscopy under local anesthesia. Utilizing a 3.7Fr Williams cystoscopic needle (Cook Urological, Spencer, IN, USA), four injections of radiotracer, each with a dose of 50 MBq and a volume of 1 mL, were accurately administered into the detrusor muscle surrounding the tumor’s edges. In the case of multiple lesions in the bladder, the injections were centered around the largest lesion. Tumor location was classified as either “left” or “right” according to a vertical midline from the bladder neck to the bladder dome. When the tumor, or tumors, exceeded the bladder axis they were denoted as “bilateral”. Hybrid SPECT-CT lymphoscintigraphy was conducted on a Gamma Camera BrightView XCT (Philips Healthcare, San Jose, CA, USA) equipped with low-energy, high-resolution collimators (LEHR) approximately 2 h after the injection. The low-dose CT projection was performed using the non-contrast option. The images acquired from SPECT and CT were fused and reconstructed utilizing Extended Brilliance Workspace V1.0 software (Philips Medical Systems Nederland B.V., Best, The Netherlands). Areas of radionuclide uptake showing markedly elevated activity compared to the background, and not topographically related to the injection site, rectum, bone marrow, kidneys, or liver, were identified as SLNs. These reconstructed images were utilized for the location of SLNs intraoperatively.

### 2.4. Definitions of Lymphadenectomy Ranges

**Figure 2:**
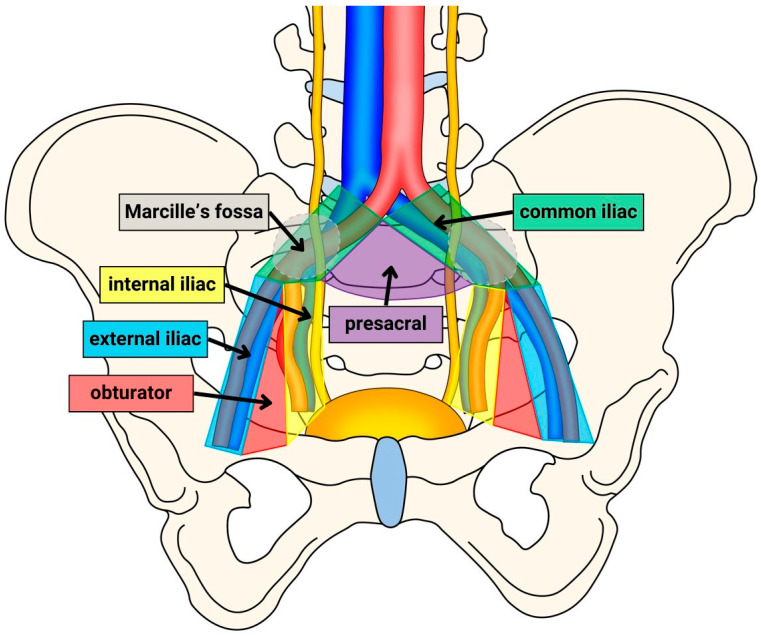
The anatomical diagram of pelvic lymph node dissection (PLND) templates divided into regions: the obturator fossa (red), external iliac vessels (blue), internal iliac vessels (yellow), common iliac vessels (green), the presacral area (purple), and the Marcille’s fossa (gray; lying behind the plane). The limited PLND (lPLND) comprises the red region; standard PLND (sPLND) includes red and blue areas; extended PLND (ePLND) covers red, blue, and yellow regions; modified-extended PLND (mePLND) covers red, blue, yellow, and purple regions; super-extended PLND (sePLND) comprises all depicted lymph node areas.

In this study, we used the following definitions for the analyzed templates of PLND: limited (lPLND), standard (sPLND), extended (ePLND), modified-extended (mePLND), and super-extended (sePLND). The lPLND includes only LNs from both obturator fossa regions. The sPLND encompasses both sides of the obturator fossa regions and external iliac regions. The ePLND involves the extraction of LNs from the obturator fossa regions and external and internal iliac regions. The mePLND includes obturator fossa regions, external and internal iliac regions, and presacral regions. Finally, the sePLND represents the resection of LNs from obturator fossa regions, internal, external, and common iliac regions, the presacral region, and Marcille’s fossa regions. The anatomical boundaries of each PLND region are described in Table 1. Figure 2 visualizes the ranges of each template.

### 2.5. Surgical Procedure

**Figure 3:**
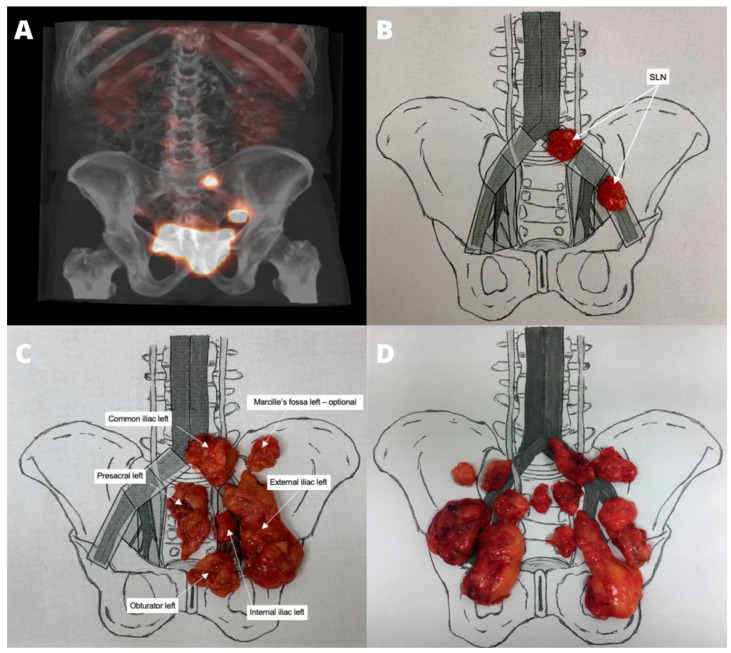
Methodology of LN mapping—presented in our previous paper [15]. (**A**) Sentinel lymph nodes (SLNs) localized using SPECT-CT images and mapped on the template (**B**); (**C**,**D**) lymphadenectomy specimens corresponding to specific anatomical areas.

In all patients, radical cystectomy, sePLND, and SLND were performed by an experienced urologist using the open technique. The fused SPECT-CT images guided the systematic scanning of all lymphatic drainage stations utilizing a handheld gamma radiation probe (FlexProbe CCXS-OP-FP, CrystalPhotonics Gmbh, Berlin, Germany). To enhance precision and minimize artifact occurrence, a probe equipped with a collimator and angled tip was used. A sustained, high-intensity radioactivity reading, at least ten times greater than the background level, was considered a positive signal. After identifying the SLNs, the targeted tissues were selectively excised and mapped on a diagram. Subsequently, sePLND was performed within the specified anatomical regions. The cranial boundary for sePLND was defined at the aortic bifurcation of the common iliac vein immediately superior to the junction of the external and internal iliac veins. The caudal boundary extended to Cooper’s ligament, with the genitofemoral nerve serving as the lateral limit. SLNs located outside the usual lymphadenectomy region were identified using SPECT-CT images, and their activity was confirmed using an intraoperative probe. In these cases, only the active tissues were removed for examination without enlarging the resection area, thereby reducing the risk of complications. The resected LNs were then mapped on a special chart. The steps are presented in Figure 3.

### 2.6. Histopathological Evaluation

The specimens were graded using the WHO 1999 and WHO 2004 systems, as well as the TNM 2010 classification system [16]. All lymphatic tissues were carefully examined as separate samples by an experienced uropathologist. After fixation, the specimens were palpated, visually examined, sectioned, stained with hematoxylin and eosin, and subsequently embedded in paraffin. To minimize the risk of laboratory errors, two identical rounds of analysis were performed. The LNs were counted and microscopically evaluated for the presence of micro-metastases (<0.2 mm) and macro-metastases (>0.2 mm), extracapsular infiltration, and cancer embolus in the lymph vessels. If malignant cells were found in the perivesical adipose tissue but lacked the typical anatomical structures of LN (capsule and subcapsular sinus), the positive lymph node status (LN+) was not reported.

### 2.7. Statistical Analysis

The binary and discrete data are displayed as counts and percentages in cross-reference tables. Continuous variables are depicted as either means and their corresponding standard deviations (SDs) or as medians along with their interquartile ranges (IQRs). Data normality was assessed using the Shapiro–Wilk test, where a *p*-value > 0.05 indicated a normal distribution. Differences among quantitative variables were appraised through the implementation of either the Student’s *t*-test or the Mann–Whitney U test. The assessment of significant differences among qualitative variables was conducted through the utilization of the Chi-square test. Additionally, Kendall’s tau-b coefficient was applied to determine if two ordinal variables can be considered statistically dependent. Confidence intervals at the 95% level were computed for estimation purposes. We assumed that the pathological examination was associated with 100% certainty in determining the LN status. Statistical significance was defined as a *p*-value < 0.05. All statistical analyses were executed utilizing Statistica v.13.3 (TIBCO Software Inc., Palo Alto, CA, USA).

## 3. Results

### 3.1. Patients Characteristics

Of the 54 participants enrolled in the research, 43 individuals (79.6%) were identified as male. The median age of the cohort was recorded as 66.9 years (range: 44–82). Eighteen patients (33.3%) were diagnosed with pT2 BCa, 19 (35.2%) with pT3 BCa, and 17 (31.5%) with pT4 BCa. On average, each patient yielded a mean of 26 harvested LNs (range: 11–50), resulting in a total of 1414 LNs being resected, which included 192 SLNs. Surgical margins were negative in all cases.

### 3.2. Topography of SLNs

All patients underwent preoperative SPECT-CT imaging and intraoperative γ-probe scanning. The anatomical distribution of SLNs was as follows: external iliac 34.4% (*n* = 66), obturator fossa 28.6% (*n* = 55), common iliac 14.1% (*n* = 27), presacral 9.4% (*n* = 18), paraaortic 5.2% (*n* = 10), pararectal 4.2% (*n* = 8), internal iliac 3.1% (*n* = 6), and paracaval 1% (*n* = 2). No SLNs were detected in the Marcille’s fossa regions. 

In 51 of 54 patients (94.4%), the number of identified hot-spot foci was identical between the SPECT-CT and γ-probe imaging. In the first of the remaining three patients, the discordance consisted of showing two hot-spot foci in SPECT-CT, while three SLNs were found intraoperatively. In another patient, eight outbreaks were recorded in the pre-operative examination, of which only seven were detected during surgery. Similarly, in a third patient, out of four imaged hot spots, one could not be located. The resection rate of SLNs was high even assuming that SLNs located outside the sePLND template were resected only if technically available (e.g., SLNs above the level of the aortic bifurcation). However, it is possible that not all SLNs were preoperatively mapped in the exact region in which they were intraoperatively located because of limited mismatches between SPECT images and intraoperative localization with the use of a γ-probe (Table 2). Nevertheless, to verify the consistency between these techniques, two statistical tests were conducted to assess agreement: McNemar’s test (*p* = 0.929) and the Wilcoxon matched-pairs signed-ranks test (*p* = 1.00). On average, 3.57 SLNs per patient were identified using SPECT-CT, with homogeneous results obtained during the intraoperative localization using the γ-probe.

### 3.3. Topography of Nodal Metastases

**Figure 4:**
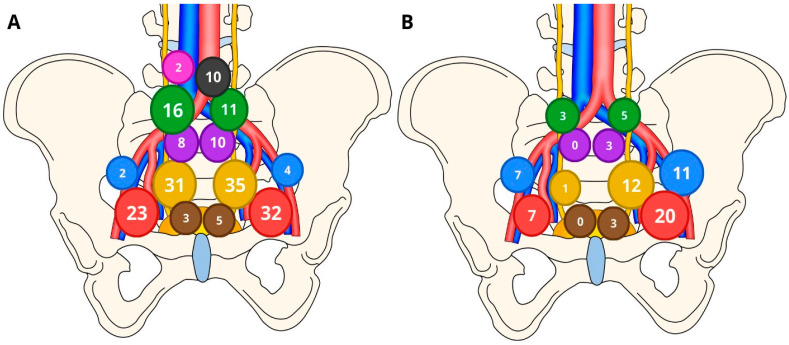
Depiction of (**A**) sentinel lymph node and (**B**) metastatic lymph node quantities. Color-coded schematics represent specific lymph node groups. Red: obturator; yellow: internal iliac; blue: external iliac; purple: presacral; green: common iliac; black: paraaortic; pink: paracaval; brown: pararectal.

A total of 72 positive LNs were found in 22 of 54 patients (41%), of whom 7 (13%) had single LN metastasis (N1) and 15 (27.8%) had multiple LN metastases (>N1). The predominant site for LN metastases was the obturator fossa region (*n* = 27/72; 37.5%) followed by the external iliac region (*n* = 18/72; 25%) and internal iliac region (*n* = 13/72; 18.1%). The remaining metastases were located in the common iliac region (*n* = 8/72; 11.1%), presacral region (*n* = 3/72; 4.2%), and pararectal region (*n* = 3/72; 4.2%). No metastases were found in the paraaortic and paracaval LNs. In 8 of 22 LN+ patients (36%), metastases did not coincide with removed SLNs and were detected only by performing sePLND. Conversely, 2 out of 22 patients (9%) exhibited LN+ detection solely through SLND and would have remained undetected by any conventional PLND template. Among the 192 SLNs examined, 24 (12.5%) showed evidence of metastasis (SLN+). Detailed topography of both SLNs and metastatic LNs is demonstrated in Figure 4.

### 3.4. Lateralization of Nodal Metastases

Among the 54 patients, 21 (38.9%) exhibited tumors exclusively on the left side, 20 (37.0%) exclusively on the right side, and 13 (24.1%) on both sides of the bladder. Regarding nodal involvement, 9 out of 21 (42.9%), 6 out of 20 (30%), and 7 out of 13 (53.8%) patients had LN metastases in the unilateral left, unilateral right, and bilateral tumor groups, respectively. In 18/22 (81.8%) of the LN+ patients, metastatic LNs were located unilaterally, with 13 patients (13/18; 72.2%) having LN metastases only on the left and 5 patients (5/18; 27.8%) having LN metastases only on the right.

In nine LN+ patients with a unilateral left tumor, eight (88.9%) had ipsilateral LN metastases and one patient (11.1%) had contralateral nodal lesions. Among six patients with a unilateral right tumor, four (66.6%) had ipsilateral LN metastases and two (33.3%) had contralateral lesions. No patient with unilateral tumor growth presented bilateral nodal metastases. For the seven patients with bilateral lesions, 3 (42.9%) had LN metastases only on the left, 1 (14.3%) only on the right, and 3 (42.9%) had bilateral nodal involvement. The intravesical lateralization of the tumor was highly correlated with the presence of ipsilateral LN metastases (Kendall’s tau-b coefficient, r = 0.58, *p* < 0.05). The inclusion of the patients with bilateral tumors in the analysis was associated with a reduction in the correlation toward medium values (r = 0.48, *p* < 0.05). Overall, 80% (12/15) of the patients with unilateral tumor growth exhibited LN metastases on the ipsilateral side of the body, while 20% (3/15) showed metastases exclusively on the contralateral side.

### 3.5. Lymphadenectomy Templates

A total of 1414 LNs were removed with the combination of sePLND and SLND. Of the 192 SLNs, 20 (10.4%) were located outside the sePLND template. Based on the pathoanatomical data, the different variants of the lymphadenectomy templates were analyzed for their effectiveness in achieving correct nodal staging in patients undergoing lymphadenectomy supplemented with the SLN technique. Performing lPLND would allow for the diagnosis of the LN+ in 50% (11/22) and the removal of all nodal metastases in 22.7% (5/22) of the nodal-affected patients. In total, 37.5% (27/72) of all metastatic LNs would be removed with lPLND. In contrast, performing sePLND would allow for the removal of 95.8% (69/72) of the affected LNs, provide correct staging for 90.9% (20/22) of the LN+ patients, and ensure that 86.4% (19/22) of the patients had all the positive LNs dissected. The use of SLND alone would allow for the detection of the LN+ in 63.8% (14/22) of the patients, the removal of all metastatic LNs in 27.3% (6/22) of the patients, and the removal of 33.3% (24/72) of metastatic LNs. The addition of SLND to the traditional PLND templates enhances the results of each template. Table 3 demonstrates the calculated values for each PLND template and the results of the possible addition of SLND.

## 4. Discussion

Accurate nodal staging is critical in patients with MIBC as it significantly impacts treatment decisions and prognostic outcomes [17,18]. Current guidelines emphasize the need to perform PLND as an integral part of surgical treatment [11,12]. This study aimed to assess the topography of LN metastases in MIBC patients using the SLN technique, analyze the side of LN metastases and primary tumor location, and evaluate the effectiveness of different PLND templates in achieving accurate LN staging.

In our study, 22 out of 54 patients (41%) were detected with LN+, yielding 72 positive LNs. The most common regions for LN invasion were the obturator fossa region (27/72; 37.5%), followed by the external iliac region (18/72; 25%), while for SLNs, the most common region was the external iliac region (66; 34.4%), followed by the obturator fossa region (55; 28.6%). We found that lymph drainage patterns and actual metastasis-related routes of BCa differ remarkably. In 9% of the LN+ cases (2 out of 22 patients), the detection of positive LNs was exclusively accomplished through SLND and would have gone unnoticed using the traditional PLND templates. All positive LNs identified beyond the scope of the conventional PLND templates were located in the pararectal region. Specifically, three instances of positive LNs in three distinct patients were observed in this anatomical area. Notably, in two of these patients, pararectal LNs were the exclusive site of positive LN detection. This underscores the potential significance of the pararectal region as a site for metastases in BCa, possibly serving as a primary location. LN metastases to the pararectal region in BCa have not been well reported. Certain researchers have included the pararectal region as a subset within larger LN groups; namely, alongside the internal iliac, presacral, or perivisceral nodes [19,20,21,22]. Nevertheless, there is a lack of studies that analyze the pararectal region as a distinct initial site for BCa metastasis. The novel revelation of unequivocal BCa-related nodal involvement in the pararectal LNs in our study suggests a noteworthy clinical implication, necessitating further investigation.

Our results revealed that combining the SLN technique with sePLND ensures the best staging outcome. Performing PLND involving only the obturator regions would allow the diagnosis of LN+ in only half of the nodal-affected patients. In contrast, sePLND facilitated the dissection of 96% of the affected LNs (69/72). However, even this extensive range of lymphadenectomy would leave 9.1% of the patients (2/22) without a proper staging. We have already discussed in our previous work that the effectiveness of the SLN technique as a standalone method is limited [15]. Nevertheless, it is noteworthy how effectively the SLN technique can improve the nodal staging of less extensive PLND templates. For instance, performing sPLND along with the SLND would yield more accurate LN+ detection compared to sePLND (95.5% vs. 90.9%). Recently, the results of the SWOG S1011 trial were presented, demonstrating that the use of sePLND instead of ePLND increases the node yield and LN+ detection [14]. However, this heightened detection rate did not translate into an improvement in overall survival (OS) and disease-free survival (DFS). Furthermore, the execution of the wider lymphadenectomy template was associated with greater morbidity and higher peri-operative mortality [14]. Meta-analyzes show better recurrence-free survival (RFS) rates in patients with more extensive PLND templates [17,23]. However, OS rates are inconsistent. The meta-analysis by Wang and Wu et al. revealed that ePLND is associated with better RFS (HR 0.74, 95% CI 0.62–0.90, *p* = 0.002) and disease-specific survival (DSS) (HR 0.66, 95% CI 0.55–0.79, *p* < 0.001) than sPLND, but not with OS (HR 0.93, 95% CI 0.55–1.58, *p* = 0.79) [18]. On the contrary, Li et al. demonstrated an association between the number of dissected LNs and favorable survival outcomes [24]. BCa patients undergoing the most extensive PLND demonstrated a 28% risk reduction corresponding to OS, a 34% risk reduction associated with cancer-specific survival (CSS), and a 36% reduction in recurrence risk when compared to patients who underwent the least extensive PLND.

It is common to compare conventional extents of PLND. A significantly less explored topic is the analysis of the feasibility of performing solely unilateral lymphadenectomy. Almost 40 years ago, Wishnow et al. conducted a single-center study on a cohort of 18 patients with unilateral bladder tumors undergoing RC, observing only ipsilateral LN metastases [25]. The hypothesis of unilateral nodal spread proposed by Wishnow was verified by May et al. using the PROMETRICS database [26]. The authors analyzed the location of LN metastases in 148 LN+ patients and demonstrated a strong association between tumor location and ipsilateral nodal spread. However, in contrast to Wishnow’s findings, as many as 33% of the patients with unilateral tumors had positive contralateral LNs. In our study, we demonstrated a robust concordance of intravesical tumor location and ipsilateralization of positive LNs. Nonetheless, 20% of the LN+ patients with unilateral tumor growth exhibited LN metastases exclusively on the opposite side to the primary tumor location. The available evidence regarding the influence of the intravesical tumor location on the topography of nodal metastases is limited. Aljabery et al. conducted an analysis on the lateralization of LN metastases concerning the tumor location within a cohort of 103 patients who underwent RC with ePLND and SLND, of whom 41 had LN+ [5]. The authors identified a statistically significant correlation between tumors located exclusively on the left or right side of the bladder and ipsilateral LN metastases. In cases of bilateral tumors, no association with the side of metastases was found. Nevertheless, akin to our study, a considerable portion of the LN+ patients (30%) presented exclusively contralateral nodal metastases. Undoubtedly, a limitation of the results lies in the small sample size. Nonetheless, the obtained findings regarding the bilateral nature of the LN metastases in MIBC reflect reports from other authors [27,28]. These findings discourage the exclusive reliance on ipsilateral PLND in the cases of distinctly unilateral tumors, despite a strong correlation for metastatic spread to the same side of the body. In two studies, Kiss et al. and Roth et al. proposed omitting the lymphadenectomy in the region of contralateral internal iliac vessels, as they did not demonstrate nodal metastases in this region. [29,30]. However, this is in contradiction with the results of Leissner et al., who obtained 7.6% of the positive LNs from the region of contralateral internal iliac vessels [31]. In our study, we documented one positive LN in this area, which casts doubt on the idea of omitting this region during PLND. The obtained results, along with available data in the literature, unequivocally indicate the necessity of performing bilateral PLND, without the possibility of selectively sparing a particular region.

The main strength of this prospective study is the fact that we used a super-wide PLND template consisting of the obturator, external, internal, and common iliac LNs, as well as of the presacral and Marcille’s fossa regions, additionally including SLNs in every patient. This wide template made it possible to cover LNs from the whole pelvis and establish which LNs are the most prone to metastasis. Another advantage is the exclusion of patients with visible LN metastases in contrast-enhanced CT or MRI. Extensive LN metastases can impair lymphatic drainage to subsequent LNs, thereby compromising proper SLN identification.

Simultaneously, this constitutes one of the main limitations of our study, namely the relatively small study population. Nonetheless, such a small population arose from our concerns regarding the potential complications posed to patients by such an extensive PLND in conjunction with the SLN identification procedure. Another limitation concerns the population characteristics. In our study, one of the inclusion criteria was ≥ pT2, while current meta-analyzes point out that the SLN technique offers the greatest efficacy in pT1 and pT2 patients and may be more suitable for patients with low-stage disease [32,33]. Moreover, a meta-analysis on the efficacy of the SLN technique revealed 79% pooled sensitivity (95% CI: 0.69–0.86%), which drops to only 70% (95% CI: 59–80%) when studies included >50% of pT3 and pT4 patients [33]. Nevertheless, the primary goal of our study was the assessment of both SLN and metastatic LN topography. Therefore, including patients with higher stages made it possible to perform a wide PLND template, and, consequently, provide a comprehensive map of lymphatic drainage. Another limitation is the lack of a separate analysis of the neoadjuvant chemotherapy (NAC) group, which was motivated by the low number of patients in this group. The meta-analysis by Zarifmahmoudi et al. showed that SLN techniques had higher sensitivity when NAC patients were excluded (82%; CI 95% 74–88%) compared to the results of pooling data from all studies (79%; 95% CI 0.69–0.86) [33]. Nevertheless, some studies have indicated that SLN detection is feasible in patients receiving NAC [34,35].

## 5. Conclusions

The pararectal region may serve as both the initial and exclusive site for LN metastases in MIBC. The obturator fossa region is the most frequent site for LN invasion, while the external iliac region serves as the primary area for SLN detection. These regions should take priority when performing PLND in MIBC patients. In the case of unilateral intravesical tumor location, there is a strong correlation with the metastatic spread to ipsilateral LNs. However, due to the possibility of contralateral metastases, the option of performing only unilateral PLND should be excluded. The most effective approach for accurate nodal staging in MIBC patients involves combining sePLND with SLND. Regardless of the chosen extent of PLND, identifying and subsequently dissecting SLNs markedly improves the efficacy of PLND. Further LN topography studies in BCa are required and should include the pararectal region.

## Figures and Tables

**Table 1:** Anatomical boundaries of lymph node dissection areas.

Region (Color in the Figure 1)	Anatomical Boarders
Obturator fossa region (red)	Bifurcation of internal and external iliac arteries, medial border of external iliac artery, pelvic floor, and obturator nerve
External iliac region (blue)	Bifurcation of internal and external iliac arteries, endopelvic fascia, psoas muscle, and medial border of external iliac artery
Internal iliac region (yellow)	Bifurcation of internal and external iliac arteries, bladder wall, obturator nerve, and pelvic floor
Common iliac region (green)	Medial boarder of common iliac artery, psoas muscle, bifurcation of internal and external iliac arteries, and aortic bifurcation
Presacral region (purple)	Medial borders of common iliac arteries and promontory and proximal sacrum to the level of bifurcation of internal and external iliac arteries
Marcille’s fossa region (grey)	The fifth lumbar vertebra medially, the inner edge of the muscle large psoas laterally, the upper edge of the wing of the sacrum below

**Table 2:** Comparison of SLN detection using preoperative SPECT-CT images and intraoperative localization with the γ-probe.

	SPECT-CT	Gamma Probe
Mean ± SD	3.6 ± 2.0	3.6 ± 1.9
Median (Q1, Q3)	3 (2, 4)	3 (2, 4)
Min; Max	0; 9	0; 9

SPECT: single-photon emission computed tomography; CT: computed tomography; SD: standard deviation; Q: quartile.

**Table 3:** The impact of the extent of lymph node dissection on correct nodal staging with the addition of radio-guided sentinel lymph node dissection.

LND Template	LN+ Patients with Correct Staging, *n*/Total (%)	LN+ Patients with All Positive LNs Removed, *n*/Total (%)	Number of Positive LNs Removed, *n*/Total (%)	Number of LNs Removed, *n*/Total (%)
lPLND	11/22 (50.0)	5/22 (22.7)	27/72 (37.5)	435/1414 (30.8)
lPLND + SLND	14/22 (63.6)	10/22 (45.5)	38/72 (52.8)	573/1414 (40.5)
sPLND	17/22 (77.3)	9/22 (40.9)	44/72 (61.1)	776/1414 (54.9)
sPLND + SLND	21/22 (95.5)	15/22 (68.2)	53/72 (73.6)	848/1414 (60.0)
ePLND	19/22 (86.4)	12/22 (54.5)	57/72 (79.2)	946/1414 (66.9)
ePLND + SLND	22/22 (100)	17/22 (77.3)	63/72 (87.5)	1012/1414 (71.6)
mePLND	19/22 (86.4)	13/22 (59.1)	61/72 (84.7)	1132/1414 (80.1)
mePLND + SLND	22/22 (100)	17/22 (77.3)	66/72 (91.7)	1180/1414 (83.5)
sePLND	20/22 (90.9)	19/22 (86.4)	69/72 (95.8)	1394/1414 (98.6)
sePLND + SLND	22/22 (100)	22/22 (100)	72/72 (100)	1414/1414 (100)
SLND only	14/22 (63.6)	6/22 (27.3)	24/72 (33.3)	192/1414 (13.6)

LN+ (positive lymph node status); SLND (sentinel lymph lode dissection); lPLND (limited pelvic lymph node dissection): obturator LNs; sPLND (standard pelvic lymph node dissection): lPLND + external iliac LNs; ePLND (extended pelvic lymph node dissection): sPLND + internal iliac LNs; mePLND (modified-extended pelvic lymph node dissection): ePLND + presacral LNs; sePLND (super-extended pelvic lymph node dissection): mePLND + Marcille’s fossa LNs + common iliac LNs.

## Data Availability

The data are available from the authors upon reasonable request.
